# Perceived stress profiles, resourcefulness, and quality of life among patients with colorectal cancer and intestinal ostomy: a cross-sectional latent profile and mediation study

**DOI:** 10.3389/fpsyt.2026.1884337

**Published:** 2026-07-29

**Authors:** Hong Wu, Li Xiao, Ling Hu, XiaoJuan Yang, Qing Wen, Liqin Liang, WenQing Guo, AiXin Zhou, Hua Zhang, Xiaocui Zou

**Affiliations:** 1 Department of Gastrointestinal Surgery, Sichuan Provincial People’s Hospital, University of Electronic Science and Technology of China, Chengdu, China; 2 Department of Geriatric Cardiology, Sichuan Provincial People’s Hospital, University of Electronic Science and Technology of China, Chengdu, China; 3 Department of Hepatobiliary and Pancreatic Surgery, Sichuan Provincial People’s Hospital, University of Electronic Science and Technology of China, Chengdu, China; 4 Department of Urology Surgery, Sichuan Provincial People’s Hospital, University of Electronic Science and Technology of China, Chengdu, China; 5 Department of Nursing, Sichuan Provincial People’s Hospital, University of Electronic Science and Technology of China, Chengdu, China; 6 Department of Gastrointestinal Surgery, The First Affiliated Hospital of Chongqing Medical University, Chongqing, China; 7 Department of Nursing, The First Affiliated Hospital of Chongqing Medical University, Chongqing, China

**Keywords:** colorectal cancer, cross-sectional study, enterostomy, latent profile analysis, mediation analysis, perceived stress, quality of life, resourcefulness

## Abstract

**Background:**

Colorectal cancer patients with intestinal ostomy often experience high levels of perceived stress, which may be associated with impaired quality of life. However, the heterogeneity of perceived stress and the underlying mechanisms linking it to quality of life remain unclear. This study aimed to identify latent profiles of perceived stress and to examine the mediating role of resourcefulness in the relationship between perceived stress profiles and quality of life among these patients.

**Methods:**

A cross-sectional study was conducted in two tertiary hospitals in the Sichuan-Chongqing region of China from February to December 2025. A total of 464 patients with colorectal cancer and intestinal ostomy completed the Perceived Stress Scale, the Resourcefulness Scale, and the Stoma-Quality of Life questionnaire. Analyses of latent profiles and mediations were performed.

**Results:**

Three perceived stress profiles were identified: Tension-Activated (31.68%), Control-Loss Dominant (52.59%), and Chronic High-Stress (15.73%). Compared with the Tension-Activated Profile, primary school education or below was a risk factor for the Chronic High-Stress Profile (OR = 6.09, 95% CI: 1.67–22.20, *P*= 0.006). Being married (OR = 0.34, 95% CI: 0.16–0.73, *P* = 0.005) and complete self-care in stoma management (OR = 0.24, 95% CI: 0.07–0.80, *P*= 0.021) were protective factors. Resourcefulness is consistent with a partial mediating role in the relationship between perceived stress profiles and quality of life (*P* < 0.001), with the indirect effects accounting for 33.2% and 36.7% of the total associations for the Control-Loss Dominant and Chronic High-Stress profile, respectively.

**Conclusions:**

Perceived stress is heterogeneous. Low education is a risk factor, whereas being married and having complete stoma self-care ability are protective factors against chronic high stress. Enhancing resourcefulness may improve quality of life.

**Clinical Trial Registration:**

http://www.chictr.org.cn/, ChiCTR2500095532.

## Background

According to statistics from the International Agency for Research on Cancer (IARC), approximately 1.93 million new cases of colorectal cancer were diagnosed worldwide in 2020, with about 0.9 million related deaths. The incidence and mortality rates ranked third and second, respectively, among all malignant tumors (1). Epidemiological data from China in 2022 showed 0.517 million new cases of colorectal cancer and 0.24 million deaths, placing the country second in cancer incidence and fourth in cancer mortality nationally (2, 3). Surgical techniques have improved greatly in recent years. However, intestinal ostomy remains an essential treatment for most patients with low rectal cancer (4). Statistics show that over 100,000 new ostomy patients are added each year in China. This number continues to grow (5). Ostomy surgery can effectively prolong patient survival. However, it also completely changes the normal pathway of fecal excretion. As a result, patients lose voluntary control over defecation. In daily life, they often face problems such as ostomy bag leakage, irregular defecation, and changes in body image. These issues trigger anxiety and inferiority. Consequently, overall quality of life is severely impaired (6).

Perceived stress refers to an individual’s subjective appraisal of stressful life events and reflects the psychological pressure and burden they experience (7). Patients with an intestinal ostomy often live under chronic high stress because of persistent physical discomfort, altered excretion patterns, social embarrassment, and role transitions after surgery (8, 9). This high-stress state is closely associated with various psychological and behavioural outcomes. Emotionally, high stress often co-occurs with an emotional inhibition coping pattern, in which patients are more likely to exhibit maladaptive psychological states such as suppression and avoidance (10). Behaviourally, patients with higher perceived stress may struggle to translate external social support into self-care ability, and their overall quality of life tends to be lower (11, 12).

In light of these associations, psychological research has provided several explanatory perspectives on individual psychological resources and coping processes. For example, some scholars have highlighted the role of coping flexibility in the stress process, suggesting that the ability to adjust coping strategies according to situational changes may significantly moderate or be associated with the relationship between perceived stress and adaptation outcomes (13). Other studies have examined coping self-efficacy and found that perceived stress is significantly correlated with coping self-efficacy and avoidant coping; patients with low self-efficacy are more likely to adopt negative coping strategies, which is associated with more severe stress experiences (14). Still, other research has focused on the potential role of resilience as a stable trait that may exert a positive effect on mental health under stressful conditions (15, 16). Collectively, these perspectives suggest that perceived stress is not a uniform experience but dynamically interacts with individual psychological resources, jointly shaping patients’ adaptation trajectories and quality of life.

However, most of the above studies have treated perceived stress as a homogeneous construct, overlooking potential heterogeneity within the patient population. Latent profile analysis (LPA) is a person−centred approach that identifies latent subgroups based on individuals’ response patterns on manifest indicators. This method enables a more accurate capture of population heterogeneity and helps to understand the relationships between perceived stress and other psychological concepts (17). Therefore, using LPA to explore the heterogeneity of perceived stress in colorectal cancer patients with intestinal ostomy holds both theoretical and practical value.

Resourcefulness is a core variable that is closely associated with perceived stress. Resourcefulness refers to an individual’s comprehensive ability to solve problems independently and seek social help, encompassing both personal resourcefulness (e.g., positive self−talk, delayed gratification, and problem−solving) and social resourcefulness (e.g., actively seeking help from others) (18). Conceptually, the dual nature of resourcefulness endows it with unique advantages in the stress−coping process: it not only helps patients rely on their own capabilities to deal with challenges but also encourages them to utilize external resources when necessary, thereby more comprehensively buffering the negative impact of stress. Empirical studies further support this association. Research has shown (19) that patients with high resourcefulness are more proactive in learning stoma care skills and actively seeking information and emotional support, thus coping more effectively with postoperative stress; conversely, those with low resourcefulness tend to resort to passive avoidance and social isolation and report significantly higher psychological distress. Another study (20) also reported that resourcefulness is consistent with a mediating role between perceived stress and depression, and may help mitigate the association between stress and mental health. Psychological interventions based on resourcefulness have been proven to effectively improve patients’ self−esteem and psychosocial adaptation while significantly reducing their perceived stress levels (21).

In summary, there is a close and stable association between resourcefulness and perceived stress, and resourcefulness may play an important mediating role between perceived stress and health outcomes. However, patients differ significantly in resourcefulness levels, and whether such differences are related to distinct profiles of perceived stress remains unclear. To address this question, the present study adopts Folkman’s Transactional Model of Stress and Coping (TMSC) as its framework (22). According to this theory, stress is not merely a stimulus or a response but a dynamic transaction between the individual and the environment (23). The model proposes two key cognitive appraisal processes: primary appraisal, which involves evaluating whether a situation is threatening or harmful, and secondary appraisal, which involves assessing one’s available coping resources and abilities to manage the situation. These appraisals collectively determine the individual’s adaptation outcomes, such as quality of life. In the context of colorectal cancer patients with intestinal ostomy, perceived stress reflects the primary appraisal of ostomy-related threats, while resourcefulness represents the secondary appraisal of personal and social coping resources (24). Therefore, based on TMSC, the present study examines the relationship between perceived stress, resourcefulness, and stoma quality of life from a person-centered perspective and tests the mediating role of resourcefulness.

**Figure 1:**
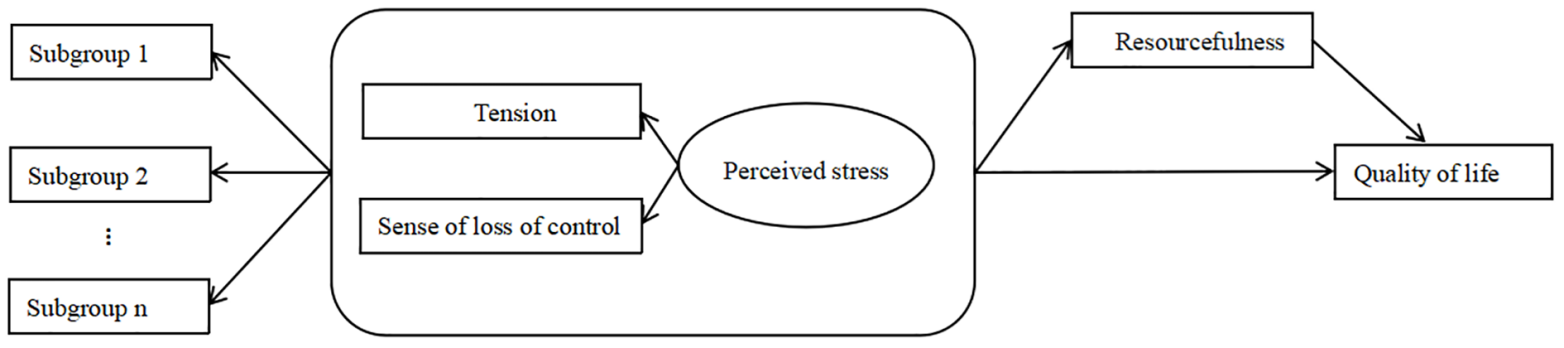
The hypothetical framework of perceived stress, resourcefulness, and quality of life among colorectal cancer and intestinal ostomy.

Based on the above theoretical and literature review, this study aims to identify heterogeneous profiles of perceived stress in colorectal cancer patients with intestinal ostomy and then further examine the mediating role of resourcefulness between different perceived stress profiles and quality of life so as to provide empirical evidence for targeted psychological interventions. Based on this, the following hypotheses are proposed (**Figure 1**): (1) Perceived stress in colorectal cancer patients with intestinal ostomy can be classified into multiple latent profiles with distinct characteristics; (2) Resourcefulness is consistent with a partial mediating role in the relationship between LPA-derived perceived stress profiles and quality of life.

## Methods

### Participants

**Figure 2:**
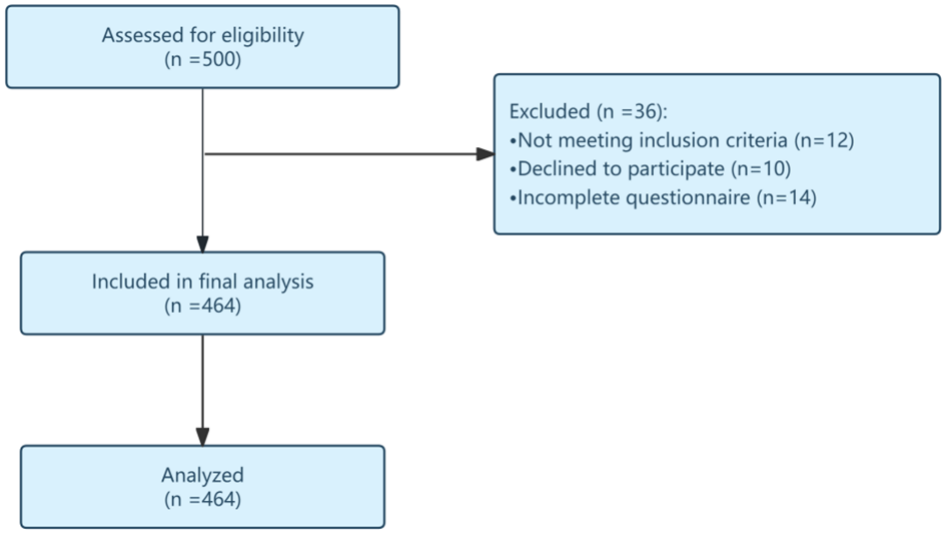
CONSORT flow diagram of patient recruitment and analysis.

An exploratory cross-sectional study was conducted in the Sichuan-Chongqing region of China between February and December 2025. Participants were recruited via convenience sampling from the gastrointestinal surgery departments of Class A tertiary-level hospitals in Chengdu and Chongqing. A review of previous LPA studies suggests that a sample size of at least 300 to 500 individuals is recommended to ensure stable and replicable solutions. Considering a 20% non-response rate, 500 participants were initially enrolled (**Figure 2**). The inclusion criteria were: (a) meeting the diagnostic criteria for colorectal cancer (CRC) with a pathologically confirmed diagnosis (25); (b) undergoing their initial ostomy surgery;(c) being aged 18 years or over; (d) being within one month post-surgery and having not yet initiated adjuvant therapy; and (e) voluntarily participating with signed informed consent. Exclusion criteria: (a) Severe psychiatric disorders or cognitive impairment; (b) Concurrent primary tumors at other sites. A total of 464 valid questionnaires were finally included, yielding a valid response rate of 92.8%.

### Procedures

Three nurses with research backgrounds and over five years of clinical experience were selected from the department as investigators. The project leader provided standardized training to all three. Before starting the survey, the investigators thoroughly explained the study to participants, obtained their consent, and collected signed informed consent forms. They then used standardized guidance language to describe the study’s purpose, content, significance, and key instructions for completing the questionnaire. During questionnaire completion, investigators promptly addressed participants’ questions without using leading language. After completion, investigators collected the questionnaires on-site and checked for completeness and logical consistency. Participants were gently reminded to check for any missing items, but were clearly informed that they could skip any question or withdraw from the study at any time without penalty. This study was approved by the hospital ethics committee (Approval No. 2022-409) and follows the principles of the Declaration of Helsinki.

### Measures

#### Demographics

Our team independently designed the sociodemographic survey questionnaire based on a systematic review of relevant domestic and international literature. It includes questions on gender, age, educational level, marital status, per capita monthly household income, occupation, type of health insurance, participation in ostomy support meetings, self-care level of stoma management, any underlying medical conditions, surgical approach, ostomy type, ostomy subtype, and any stoma complications. Patients completed the questionnaire independently, and the data were verified against electronic medical records.

#### Measurement of stress

The Perceived Stress Scale (PSS), developed by Cohen et al. (26), is designed to assess how individuals perceive their lives as stressful over the preceding month. This study employed the Chinese version of the Perceived Stress Scale (CPSS-14), which was translated and revised by Yang et al. (27). The scale consists of 14 items across two dimensions: the Tension dimension (items 1, 2, 3, 8, 11, 12, and 14) and the Sense of Loss of Control dimension (items 4, 5, 6, 7, 9, 10, and 13). Responses are recorded on a 5-point Likert scale ranging from 0 (“never”) to 4 (“very often”). Items 4, 5, 6, 7, 9, 10, and 13 are positively worded but are reverse-scored. The total score ranges from 0 to 56; a higher score indicates a greater level of perceived stress. The reported Cronbach’s α for the original scale is 0.78 (27). In the present study, the Cronbach’s α was 0.912.

#### Measurement of resourcefulness

The Resourcefulness Scale (RS), developed by Zauszniewski et al. (28), assesses an individual’s level of resourcefulness. This study employed the Chinese version of the Resourcefulness Scale (RS-C) by Wang Shumi et al. (29). The scale comprises 28 items across two dimensions: personal and social resourcefulness. Sixteen of these items measure personal resourcefulness, while the remaining 12 items assess social resourcefulness. The instrument uses a 6-point Likert scale, with response options ranging from Level 1 (‘Strongly Disagree’) to Level 6 (‘Strongly Agree’). Scoring is coded from 0 to 5 (Level 1 = 0, Level 2 = 1, Level 3 = 2, Level 4 = 3, Level 5 = 4, Level 6 = 5). All 28 items are positively worded and scored in the same direction; no reverse-scored items are included in the scale. Total scores range from 0 to 140, with higher scores indicating greater resourcefulness. The scale’s Cronbach’s α is 0.85 (29). In this study, the Cronbach’s α for the scale was 0.965, indicating excellent internal consistency, though the high value may also reflect a degree of item covariance.

#### Measurement of quality of life

The Stoma-Quality of Life (Stoma-QOL) questionnaire was developed by Prieto et al. to comprehensively and sensitively measure the quality of life of patients with a colostomy or ileostomy, based on Hunt and McKenna’s basic QOL demand model (30). This study used the Chinese version of the Stoma-QOL (Stoma-QOL-C), which was translated and culturally adapted by Shao et al. (31). The scale comprises four dimensions: social relationship, psychological impact, defecation concern, and daily function, and contains a total of 20 items. It uses a 4-point Likert scale, where 1 represents “always,” 2 represents “sometimes,” 3 represents “rarely,” and 4 represents “never.” The total score is calculated by summing the scores of all items, with a possible range of 20 to 80 points. The Cronbach’s α coefficient for the Stoma-QOL-C was 0.935 (31). In this study, the Cronbach’s α for the scale was 0.949.

### Data analyses

Data were analyzed using SPSS 22.0, Mplus 8.3, and JASP 0.16.0, with statistical significance set at *P* < 0.05. Since data were collected via self-reported questionnaires at a single time point, Harman’s single-factor test was applied to assess common method bias. The first unrotated factor accounted for 37.50% of the total variance, suggesting that common method bias may not be a severe concern in this dataset.

First, descriptive statistics were computed. Categorical variables were summarized using frequencies and percentages, while continuous variables were expressed as means and standard deviations. To examine subgroup differences, normality and homogeneity of variances were assessed using the Kolmogorov-Smirnov and Levene’s tests, respectively. Independent samples t-tests or one-way ANOVA were applied for normally distributed data with equal variances, whereas the Mann–Whitney U or Kruskal-Wallis H tests were used for two or more groups, respectively, when assumptions were violated.

Second, Pearson correlation analysis was used to examine bivariate associations among perceived stress, resourcefulness, and quality of life.

Third, latent profile analysis (LPA) was conducted to identify perceived stress-based subgroups. To ensure robust profiles, sensitivity analyses involved re-running the LPA with different starting values and checking for outliers. Models with one to five latent classes were estimated and compared using AIC, BIC, and sample-size-adjusted BIC (aBIC), where lower values indicated better fit. The LMRT and BLRT tests were also applied, with a significant *P* < 0.05 indicating that the k-class model outperformed the (k-1)-class model. Classification accuracy was evaluated using entropy, with values closer to 1 (≥ 0.8, reflecting ≥ 90% accuracy) suggesting clearer profile separation. The selected model balanced goodness of fit, parsimony, and interpretability (32, 33). Factors associated with LPA-derived perceived stress were analyzed using univariable and multivariable logistic regression (34).

Fourth, differences in resourcefulness and quality-of-life scores across LPA-derived perceived stress profiles were examined using Analysis of Variance (ANOVA), followed by Bonferroni-corrected *post hoc* tests to identify specific pairwise differences.

Finally, mediation analysis was conducted using PROCESS Model 4 (5,000 bootstrap resamples) to examine the mediating role of resourcefulness in the relationship between perceived stress profiles and quality of life. Covariates demonstrating significant associations were included as controls in both the mediator and outcome equations across all models. Perceived stress profiles were dummy-coded with the Tension-Activated Profile (C1) as the reference group.

### Ethical considerations

Our study was conducted in accordance with the principles outlined in the Declaration of Helsinki. All the procedures performed in this study were approved by the Medical Ethics Committee of Sichuan Provincial Academy of Medical Sciences-Sichuan Provincial People’s Hospital (Approval No. 2022-409). All participants provided written informed consent for the use of their data in non-commercial scientific research. All participants were informed of their right to withdraw from the study at any time without negative consequences. This study exclusively involved human participants.

## Results

### Demographic and clinical characteristics of participants with perceived stress scores stratified by each variable

A total of 464 patients with colorectal cancer and intestinal ostomy were included (mean age 63.90 ± 11.66 years; 66.81% male). As shown in **Table 1**, significant differences in perceived stress scores were observed across several variables.

**Table 1:** Demographic and clinical characteristics of participants with perceived stress scores stratified by each variable (N = 464).

Variable	Category	n (%)	Perceived stress (M ± SD)	Statistic	P
Gender	Male	310 (66.81)	28.41 ± 9.53	1.578a	0.115
	Female	154 (33.19)	26.93 ± 9.55		
Age group	≤60 years	183 (39.44)	27.26 ± 9.14	-1.208a	0.228
	>60 years	281 (60.56)	28.35 ± 9.81		
Educational level	Primary school or below	122 (26.29)	34.40 ± 10.33	79.789c	**<0.001**
	Junior high school	127 (27.37)	27.72 ± 7.70		
	High school/technical secondary	141 (30.39)	25.14 ± 6.57		
	College degree or above	74 (15.95)	22.88 ± 10.30		
Marital status	Married	278 (59.91)	25.02 ± 8.08	-8.596a	**<0.001**
	Unmarried/divorced/widowed	186 (40.09)	32.25 ± 9.95		
Per capita monthly household income (CNY)	<3000	145 (31.25)	28.60 ± 9.98	24.183b	**<0.001**
	3000–4999	207 (44.61)	30.19 ± 8.78		
	≥5000	112 (24.14)	22.85 ± 8.51		
Occupation	Employed	258 (55.60)	27.60 ± 9.31	0.505b	0.604
	Unemployed	77 (16.59)	27.82 ± 10.48		
	Retired	129 (27.80)	28.63 ± 9.50		
Health insurance type	Self-paid	37 (7.97)	30.35 ± 8.75	2.001b	0.113
	New Rural Cooperative Medical Scheme	172 (37.07)	27.46 ± 9.71		
	Urban-Rural Resident Basic Medical Insurance	154 (33.19)	28.74 ± 10.04		
	Employee Medical Insurance	101 (21.77)	26.56 ± 8.62		
Participation in ostomy support meetings	No	243 (52.37)	30.60 ± 9.47	6.613a	**<0.001**
	Yes	221 (47.63)	24.98 ± 8.76		
Self-care level of stoma management	Complete self-care	104 (22.41)	22.98 ± 8.89	63.711c	**<0.001**
	Partial self-care	220 (47.41)	26.75 ± 7.24		
	Completely dependent on others	140 (30.17)	33.44 ± 10.57		
Underlying medical conditions	No	297 (64.01)	26.19 ± 8.35	-5.011a	**<0.001**
	Yes	167 (35.99)	31.01 ± 10.74		
Surgical approach	Open surgery	55 (11.85)	28.67 ± 10.45	0.622a	0.534
	Laparoscopic surgery	409 (88.15)	27.82 ± 9.44		
Ostomy type	Temporary	348 (75.00)	26.81 ± 9.25	-4.420a	**<0.001**
	Permanent	116 (25.00)	31.25 ± 9.71		
Ostomy subtype	Colostomy	228 (49.14)	28.09 ± 9.13	0.371a	0.711
	Ileostomy	236 (50.86)	27.76 ± 9.96		
Stoma complications	No	397 (85.56)	26.79 ± 8.33	-4.753a	**<0.001**
	Yes	67 (14.44)	34.63 ± 13.06		

a, *t*-value; b, *F*-value; c, *H*-value. M, mean; SD, standard deviation. Bold figures indicate statistically significant results (*P* < 0.05).

Among the variables analyzed, participants with lower educational levels reported higher perceived stress scores (*H* = 79.789, *P* < 0.001). Furthermore, unmarried/divorced/widowed participants had higher stress scores than married participants (*t* = -8.596, *P* < 0.001). In addition, participants with a monthly household income of 3000–4999 CNY reported the highest stress scores (*F* = 24.183, *P* < 0.001).

Similarly, individuals who did not attend ostomy support meetings had higher stress scores than those who attended (*t* = 6.613, *P* < 0.001). Participants who completely relied on others for stoma care had the highest stress scores (33.44 ± 10.57), followed by those who partially relied on others (26.75 ± 7.24) and those who practiced complete self-care (*H* = 63.711, *P* < 0.001). Additionally, higher stress scores were observed among participants with underlying medical conditions (*t* = -5.011, *p* < 0.001), a permanent ostomy (*t* = -4.420, *p* < 0.001), and stoma complications (*t* = -4.753, *p* < 0.001). In contrast, no significant differences were found for gender, age, occupation, insurance type, surgical approach, or stoma type (all *P* > 0.05).

### Correlation analysis among perceived stress, resourcefulness, and quality of life

**Figure 3:**
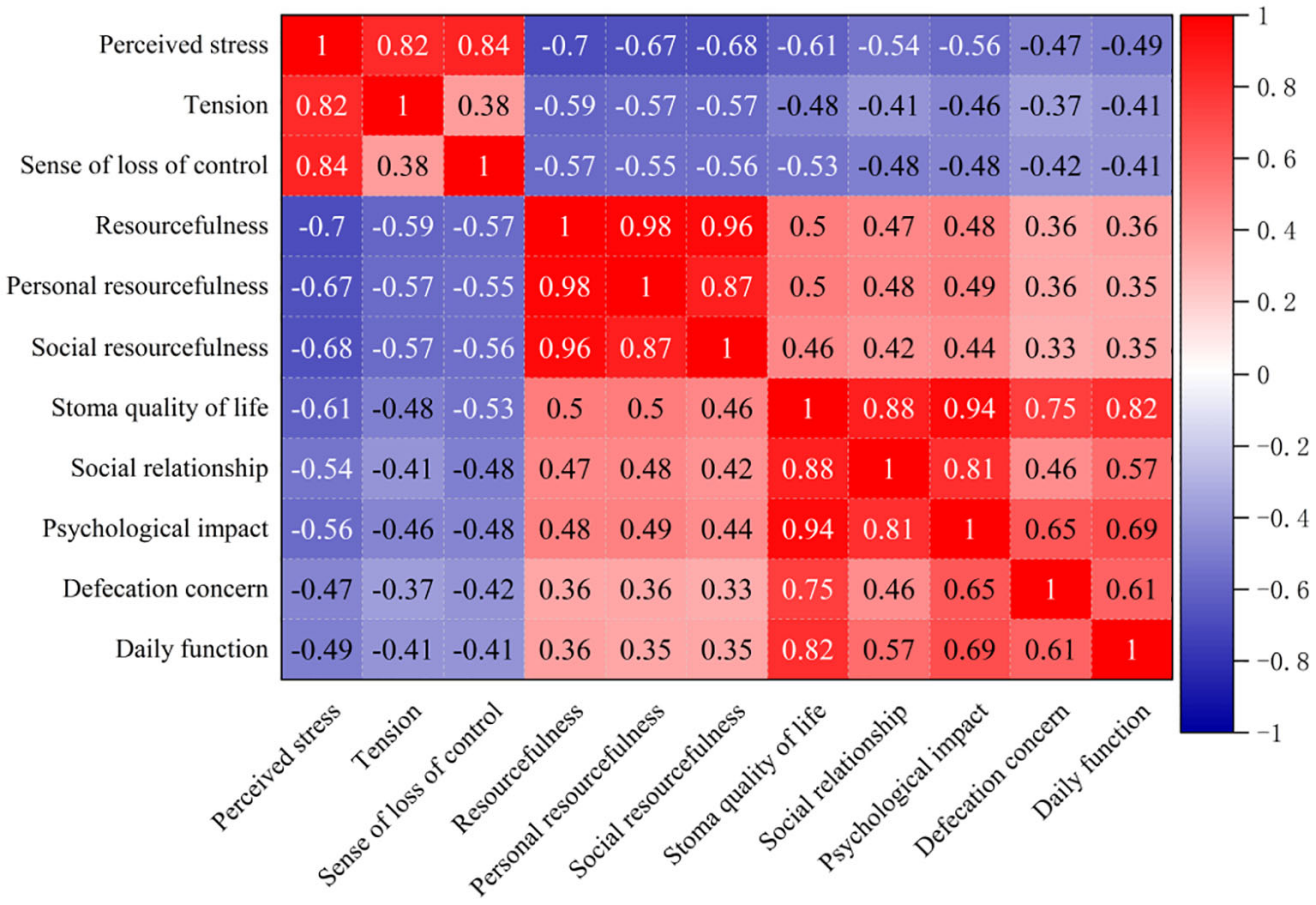
Pearson correlation heatmap of perceived stress, resourcefulness, and quality of life. C1 = Tension-Activated Profile; C2 = Control-Loss Dominant Profile; C3 = Chronic High-Stress Profile.

The mean and standard deviations of variables were perceived stress (27.92 ± 9.55), resourcefulness (85.93 ± 21.92), and stoma quality of life (46.39 ± 11.90). **Figure 3** illustrates Pearson’s correlation heatmap (all *P* < 0.001). Perceived stress was negatively associated with resourcefulness (*r* = −0.697) and stoma quality of life (*r* = −0.609). Resourcefulness was positively associated with stoma quality of life (*r* = 0.500). These significant correlations provided a foundation for subsequent mediation analysis.

### Latent profiles analysis of perceived stress

We performed latent profile analysis (LPA) to identify subgroups of perceived stress. **Table 2** shows the fit indices for one− to five−class models. Model selection was based on a comprehensive evaluation of statistical fit, parsimony, interpretability, and clinical utility. While the AIC, BIC, and aBIC continued to decrease from the three- to five-class solutions, the Lo-Mendell-Rubin Likelihood Ratio Test (LMRT) became non-significant for the four-class model (p = 0.0728), favoring the three-class solution. Although the Bootstrap Likelihood Ratio Test (BLRT) remained significant, the three-class solution was ultimately selected based on a combination of statistical parsimony (supported by LMRT), high entropy (0.941), adequate and clinically interpretable class sizes (all > 10%), and the clear theoretical meaningfulness and clinical utility of the three distinct profiles identified. Classification accuracy was further evaluated using average posterior probabilities for the most likely class membership, which were 0.972, 0.977, and 0.982 for Class 1, 2, and 3, respectively, indicating highly accurate profile assignment. Hypothesis 1 was supported.

**Table 2:** Fit indices and group sizes of latent profile analysis models (N = 464).

Indicators	1-Class	2-Class	3-Class	4-Class	5-Class
Fit statistics					
AIC	18475.955	16949.766	**15901.871**	15219.573	14860.800
BIC	18591.871	17127.781	**16141.984**	15521.785	15225.110
aBIC	18503.006	16991.310	**15957.906**	15290.101	14945.820
Entropy	—	0.951	**0.941**	0.927	0.939
LMRT (P)	—	<0.001	**0.001**	0.0728	0.0589
BLRT (P)	—	<0.001	**<0.001**	<0.001	<0.001
Group size (%)					
C 1	100	80.82	**31.68**	21.77	18.53
C 2	—	19.18	**52.59**	19.40	40.09
C 3	—	—	**15.73**	42.89	6.68
C 4	—	—	—	15.94	18.53
C 5	—	—	—	—	16.16

AIC, Akaike information criterion; BIC, Bayesian information criterion; aBIC, adjusted BIC; LMRT, Lo–Mendell–Rubin likelihood ratio test; BLRT, bootstrap likelihood ratio test. Bold values indicate the selected class solution.

**Figure 4:**
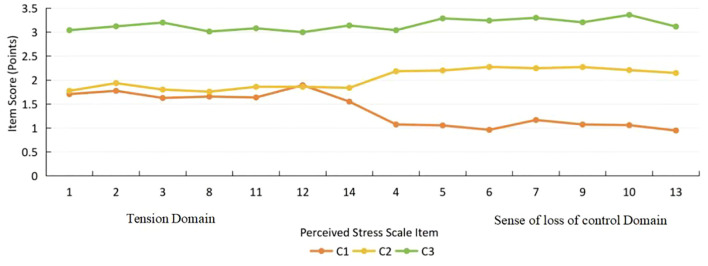
Parameters for the final three-class patterns. The three latent profiles derived from latent profile analysis (LPA) of perceived stress were set as the independent variable (X), with the Tension-Activated Profile specified as the reference group. Resourcefulness served as the mediator (M), and stoma quality of life was defined as the dependent variable (Y). All path coefficients presented in the figure are standardized values. The mediation model was fully adjusted for the following pre-specified covariates: educational level, marital status, per capita monthly household income participation in ostomy support meetings, self-care level of stoma management, underlying medical conditions, ostomy type (temporary vs. permanent), and stoma complications. The control variables are not presented in the figure for brevity. **P < 0.001, *P < 0.01.

**Figure 4** displays the three perceived stress profiles. Class 1 was labeled the Tension-Activated Profile (n = 147, 31.68%), characterized by high tension scores but low loss-of-control scores. Class 2 was labeled the Control-Loss Dominant Profile (n = 244, 52.59%), showing prominent loss of control alongside relatively low tension. Class 3 was labeled the Chronic High-Stress Profile (n = 73, 15.73%), with consistently elevated scores on both dimensions.

Categorical covariates were coded for regression analysis as follows: Educational level was ordinal-coded from 1 (Primary school or below) to 4 (College degree or above), with college degree serving as the reference. Marital status was dummy-coded as 0 (Married, reference) and 1 (Unmarried/divorced/widowed). Per capita monthly household income was categorized into three levels: 1 (<3000 CNY), 2 (3000–4999 CNY), and 3 (≥5000 CNY), with the highest income bracket (≥5000 CNY) set as the reference. Participation in ostomy support meetings was coded as 0 (Yes, reference) and 1 (No). Self-care level was coded ordinally: 1 (Completely dependent on others, reference), 2 (Partial self-care), and 3 (Complete self-care). Regarding clinical factors, underlying medical conditions were coded as 0 (Yes, reference) and 1 (No); ostomy type as 0 (Permanent, reference) and 1 (Temporary); and stoma complications as 0 (Present, reference) and 1 (Absent). To assess potential multicollinearity in the regression models, variance inflation factors (VIFs) were computed for all predictors. All VIF values were below 2.0 (range: 1.107-1.512), indicating that multicollinearity was not a substantial concern in the adjusted models. Multivariate logistic regression (see **Table 3**) showed that, compared with the Tension-Activated Profile, primary school education or below was a risk factor for the Chronic High-Stress Profile (OR = 6.09, 95% CI: 1.67–22.20, *P* = 0.006). Being married was a protective factor (OR = 0.34, 95% CI: 0.16–0.73, *P* = 0.005), and complete self-care in stoma management was also a protective factor (OR = 0.24, 95% CI: 0.07–0.80, *P* = 0.021). For the comparison between the Control-Loss Dominant Profile and the Tension-Activated Profile, partial self-care (OR = 1.98, 95% CI: 1.08-3.62, P = 0.027) was a significant predictor.

**Table 3:** Univariate and multivariate logistic regression results for predicting external features of the three-class pattern.

	Univariate analysis				Multivariate analysis			
Variables	C2vsC1		C3vsC1		C2vsC1		C3vsC1	
	OR (95%CI)	P	OR (95%CI)	P	OR (95%CI)	P	OR (95%CI)	P
Primary school or below (ref. college or above)	2.22 (1.12-4.38)	**0.022**	18.26 (5.80-57.54)	**<0.001**	1.47 (0.68-3.21)	0.329	6.09 (1.67-22.2)	**0.006**
Junior high school (ref. college or above)	2.26 (1.22-4.17)	**0.009**	3.25 (0.97-10.95)	0.057	1.7 (0.87-3.29)	0.119	1.98 (0.53-7.4)	0.312
Married (ref. unmarried/divorced/widowed)	0.55 (0.35-0.86)	**0.009**	0.13 (0.07-0.25)	**<0.001**	0.64 (0.39-1.07)	0.086	0.34 (0.16-0.73)	**0.005**
Per capita monthly household income <3000 CNY (ref. ≥5000 CNY)	1.72 (1.00-2.98)	0.051	5.93 (2.22-15.82)	**<0.001**	1.49 (0.82-2.72)	0.193	1.26 (0.40-3.96)	0.695
Per capita monthly household income 3000–4999 CNY (ref. ≥5000 CNY)	1.33 (0.81-2.18)	0.261	4.85 (1.90-12.40)	**0.001**	0.91 (0.52-1.60)	0.739	0.78 (0.25-2.37)	0.655
Participation in ostomy support meetings (ref. yes)	1.58 (1.05-2.39)	**0.030**	3.74 (2.03-6.88)	**<0.001**	1.33 (0.80-2.20)	0.267	1.43 (0.66-3.11)	0.371
Complete stoma self-care (ref. completely dependent on others)	0.56 (0.31-1.00)	0.050	0.06 (0.02-0.18)	**<0.001**	0.86 (0.42-1.79)	0.690	0.24 (0.07-0.80)	**0.021**
Partial stoma self-care (ref. completely dependent on others)	1.44 (0.85-2.43)	0.172	0.19 (0.09-0.37)	**<0.001**	1.98 (1.08-3.62)	0.027	0.46 (0.2-1.04)	0.062
Underlying medical conditions (ref. yes)	0.63 (0.40-0.99)	**0.045**	0.26 (0.14-0.47)	**<0.001**	0.65 (0.39-1.07)	0.091	0.53 (0.25-1.12)	0.095
Temporary ostomy (ref. permanent ostomy)	0.68 (0.41-1.12)	0.130	0.36 (0.19-0.68)	**0.002**	0.78 (0.45-1.35)	0.378	0.83 (0.38-1.80)	0.637
Stoma complications (ref. yes)	1.17 (0.60-2.30)	0.641	0.2 (0.10-0.40)	**<0.001**	1.35 (0.65-2.82)	0.424	0.52 (0.22-1.25)	0.141

C1, Tension-Activated Profile; C2, Control-Loss Dominant Profile; C3, Chronic High-Stress Profile. Only variables that showed statistically significant associations (*P* < 0.05) in either univariate or multivariate analyses are presented in this table. Non-significant variables were omitted for clarity and conciseness. Bold figures indicate statistically significant results (*P* < 0.05).

### LPA-based perceived stress differences in resourcefulness and quality of life scores

As shown in **Table 4**, significant between-group differences were observed across the three perceived stress profiles in resourcefulness and stoma quality-of-life scores. For total resourcefulness, the Chronic High-Stress Profile (C3) scored the lowest (58.31 ± 21.95), followed by the Control-Loss Dominant Profile (C2, 85.17 ± 16.10), and the Tension-Activated Profile (C1) scored the highest (100.91 ± 15.71), with a significant overall difference (*F* = 153.01, *P* < 0.001). Regarding stoma quality of life, the Chronic High-Stress Profile again reported the lowest scores (35.19 ± 11.56), followed by the Control-Loss Dominant Profile (45.74 ± 8.73) and the Tension-Activated Profile (53.02 ± 12.17), with a significant overall difference (*F* = 72.78, *P* < 0.001). *Post-hoc* comparisons revealed a consistent stepwise pattern: C1 > C2 > C3 for total resourcefulness, personal resourcefulness, social resourcefulness, total stoma quality of life, and all four subdomains (social relationship, psychological impact, defecation concern, and daily function).

**Table 4:** ANOVA comparisons of resourcefulness and stoma quality of life scores across LPA-based perceived stress profiles and *post-hoc* comparisons.

Variable	C1 (n=147)	C2 (n=244)	C3 (n=73)	F	P	Post-hoc
**Resourcefulness (total)**	100.91 ± 15.71	85.17 ± 16.10	58.31 ± 21.95	153.01	**<0.001**	C1>C2>C3
Personal resourcefulness	58.52 ± 9.48	49.40 ± 9.74	34.64 ± 13.39	131.39	**<0.001**	C1>C2>C3
Social resourcefulness	42.38 ± 7.18	35.77 ± 7.21	23.67 ± 9.79	145.55	**<0.001**	C1>C2>C3
**Stoma quality of life (total)**	53.02 ± 12.17	45.74 ± 8.73	35.19 ± 11.56	72.78	**<0.001**	C1>C2>C3
Social relationship	17.52 ± 4.24	15.06 ± 3.50	11.78 ± 5.32	49.70	**<0.001**	C1>C2>C3
Psychological impact	15.97 ± 3.92	14.00 ± 3.03	10.74 ± 3.79	56.13	**<0.001**	C1>C2>C3
Defecation concern	9.54 ± 2.75	8.25 ± 2.24	6.32 ± 2.19	44.47	**<0.001**	C1>C2>C3
Daily function	9.98 ± 3.35	8.43 ± 2.21	6.36 ± 2.47	46.16	**<0.001**	C1>C2>C3

C1, Tension-Activated Profile; C2, Control-Loss Dominant Profile; C3, Chronic High-Stress Profile. Data are presented as mean ± standard deviation. *Post-hoc* comparisons were conducted using Bonferroni correction. Bold figures indicate statistically significant results (*P* < 0.05).

### Mediation analysis of resourcefulness between LPA-based perceived stress and quality of life

The mediation analysis was performed using Hayes’ PROCESS Model 4, with all covariates that demonstrated significant associations with key study variables in **Table 1** incorporated into both the mediator (resourcefulness) and outcome (stoma quality of life) equations. Specifically, the adjusted covariates included educational level, marital status, monthly household income, participation in ostomy support meetings, self-care level of stoma management, underlying medical conditions, ostomy type (temporary vs. permanent), and stoma complications. The results indicated that resourcefulness exerted a statistically significant indirect association between perceived stress profiles and stoma quality of life. As shown in **Tables 5**, **6**, the Tension-Activated Profile (C1) served as the reference group. The indirect effect of the Control-Loss Dominant Profile (C2) on stoma quality of life through resourcefulness was -2.256 (95% CI: -3.356 to -1.297); the direct effect was -4.533 (95% CI: -6.748 to -2.317); and the total effect was -6.789 (95% CI: -8.942 to -4.636). The mediating effect accounted for 33.2% of the total effect.

**Table 5:** The mediating effect of perceived stress profiles on stoma quality of life.

Outcome: resourcefulness	β	SE	P	95% CI
C2 (vs. C1)	-13.598	1.739	**<0.001**	-17.015, -10.181
C3 (vs. C1)	-34.138	2.660	**<0.001**	-39.365, -28.911
Educational level	1.962	0.886	**0.027**	0.220, 3.703
Marital status	-2.628	1.737	0.131	-6.042, 0.785
Monthly income	2.064	1.084	0.058	-0.067, 4.195
Attended support meetings	2.280	1.763	0.197	-1.186, 5.745
Self-care level of stoma management	-2.830	1.309	**0.031**	-5.404, -0.257
Underlying conditions	-2.743	1.735	0.115	-6.153, 0.667
Ostomy type (temporary)	0.280	1.841	0.879	-3.338, 3.898
Stoma complications	-2.186	2.368	0.356	-6.840, 2.467
Outcome: quality of life				
C2 (vs. C1)	-4.533	1.127	**<0.001**	-6.748, -2.317
C3 (vs. C1)	-9.789	1.890	**<0.001**	-13.503, -6.074
Resourcefulness	0.166	0.029	**<0.001**	0.110, 0.222
Educational level	-0.144	0.542	0.791	-1.210, 0.922
Marital status	-1.164	1.060	0.273	-3.247, 0.919
Monthly income	-0.282	0.662	0.670	-1.584, 1.020
Attended support meetings	0.877	1.075	0.415	-1.236, 2.990
Self-care level of stoma management	-0.602	0.801	0.453	-2.176, 0.972
Underlying conditions	2.641	1.059	**0.013**	0.559, 4.722
Ostomy type (temporary)	-2.340	1.120	**0.037**	-4.541, -0.138
Stoma complications	-1.234	1.442	0.393	-4.068, 1.601

C1, Tension-Activated Profile; C2, Control-Loss Dominant Profile; C3, Chronic High-Stress Profile. CI, confidence interval. R², 0.459 for the resourcefulness model, and 0.321 for the quality of life model. Bold figures indicate statistically significant results (*P* < 0.05).

**Table 6:** Direct and indirect effects of perceived stress profiles on stoma quality of life.

Variables	Effect	SE	LLCI	ULCI
Direct effect				
Control-Loss Dominant Profile (C2)	-4.533	1.127	-6.748	-2.317
Chronic High-Stress Profile (C3)	-9.789	1.890	-13.503	-6.074
Indirect effect				
Control-Loss Dominant Profile (C2)	-2.256	0.526	-3.356	-1.297
Chronic High-Stress Profile (C3)	-5.664	1.132	-7.959	-3.468
Total effect				
Control-Loss Dominant Profile (C2)	-6.789	1.096	-8.942	-4.636
Chronic High-Stress Profile (C3)	-15.453	1.676	-18.746	-12.160

C1 (Tension-Activated Profile) is the reference category. LLCI, lower limit of 95% confidence interval; ULCI, upper limit of 95% confidence interval.

For the Chronic High-Stress Profile (C3), the indirect effect through resourcefulness was -5.664 (95% CI: -7.959 to -3.468), the direct effect was -9.789 (95% CI: -13.503 to -6.074), and the total effect was -15.453 (95% CI: -18.746 to -12.160). The mediating effect accounted for 36.7% of the total effect.

**Figure 5:**
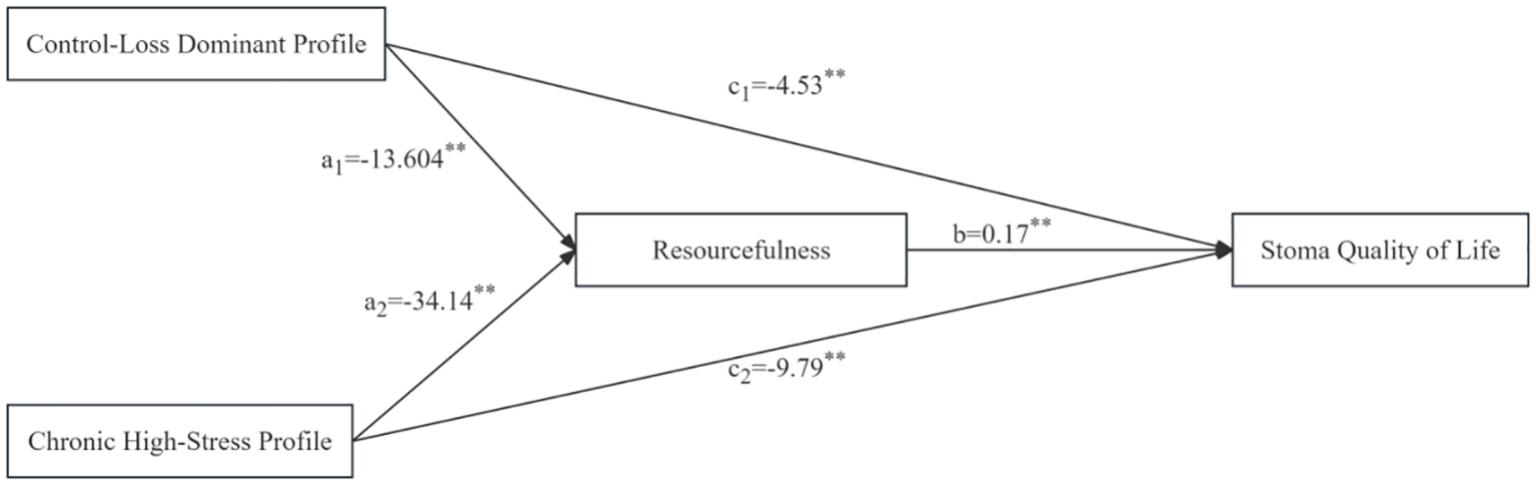
Hypothesized mediator model.

These results indicate that resourcefulness is consistent with a partial mediating role in the relationship between perceived stress profiles and stoma quality of life. The mediation model is illustrated in **Figure 5**. Thus, Hypothesis 2 was supported.

## Discussion

This study used latent profile analysis to identify three perceived stress profiles among colorectal cancer patients with an intestinal ostomy: Tension-Activated Profile (31.68%), Control-Loss Dominant Profile (52.59%), and Chronic High-Stress Profile (15.73%). Tension-Activated Profile patients had high tension but low loss of control, indicating high physiological arousal with relatively positive cognitive appraisal. Control-Loss Dominant Profile patients showed a prominent loss of control with relatively low tension, suggesting that lack of control is their main stressor. Chronic High-Stress Profile patients had persistently high scores on both dimensions, suggesting a pattern consistent with long-term high stress load and associated psychological resource depletion. These results align with Liang et al. (35), further supporting the heterogeneity of perceived stress in this population. Chronic High-Stress Profile patients require more clinical attention because they had the lowest scores in resourcefulness and quality of life among the three groups, and a high proportion of them were completely dependent on others for stoma care. Multivariate logistic regression showed that independent predictors of the Chronic High-Stress Profile (compared to the Tension-Activated Profile) included primary school education or below as a risk factor, being married as a protective factor, and complete self-care as a protective factor. Patients with low education have insufficient health literacy and struggle to master stress management and stoma care skills, which increases their risk of chronic high stress (36). In contrast, married patients benefit from spousal emotional support and daily care, which helps buffer stress (37). Similarly, patients who are capable of complete stoma self-care maintain a sense of bodily control and higher self-efficacy, thereby reducing their vulnerability to chronic high stress (38). However, it should be noted that poor self-care ability may reflect underlying disease severity or functional limitations rather than merely a manifestation of stress status; therefore, when interpreting this predictor clinically, objective indicators such as tumour stage and performance status should be considered together. These vulnerability characteristics provide clear indicators for early clinical screening and stratified intervention.

This study showed that resourcefulness (total, personal, and social) and stoma quality of life (total and four dimensions) decreased stepwise across the three perceived stress profiles (C1 > C2 > C3, all *P* < 0.001). These findings demonstrate a stepwise association across the three perceived stress profiles, with higher stress profiles showing lower resourcefulness and quality of life scores. However, the directionality of these associations remains open to interpretation: lower quality of life may exacerbate stress perception, and resourcefulness could be either a determinant or a consequence of chronic stress. Therefore, the observed negative correlations should not be unequivocally interpreted as causal effects of perceived stress on resourcefulness and quality of life. Tension-Activated Profile patients had the highest resourcefulness and quality-of-life scores because they actively mobilized personal and social resources to cope with postoperative challenges (35). Chronic High-Stress Profile patients had the lowest scores. Long-term tension and loss of control were significantly associated with psychological resource depletion in this group and were accompanied by lower problem-solving ability and help-seeking willingness. Consequently, they had insufficient stoma care skills, social avoidance, and increased defecation concerns, which ultimately impaired their overall quality of life (39). Regarding the daily function dimension, no significant difference was found between the Tension-Activated Profile and the Control-Loss Dominant Profile, but both were significantly higher than the Chronic High-Stress Profile. This finding suggests that impaired daily function may be a “threshold feature” of the Chronic High-Stress Profile, meaning that behavioral restrictions only emerge when stress accumulates to a certain level (40). Clinical observations suggest that high-stress patients often voluntarily reduce going out and social activities due to fear of stoma bag leakage or odor, and this behavioral inhibition is associated with decreased daily function (41, 42).

This study found that resourcefulness played an important mediating role between perceived stress profiles and stoma quality of life. Our hypothesized framework was accepted (**Figure 1**). For the Control-Loss Dominant Profile and Chronic High-Stress Profile (compared with the Tension-Activated Profile), the indirect effects accounted for 33.2% and 36.7% of the total effects, respectively. The mechanism of this partial mediation can be attributed to two factors: the negative effect of perceived stress on resourcefulness and the positive effect of resourcefulness on quality of life. First, persistent tension and loss of control were significantly correlated with lower levels of their willingness and ability to solve problems independently and seek social help (43). In this study, chronic high-stress patients scored significantly lower than tension-activated patients in both personal and social resourcefulness, confirming the negative association between high stress and low psychological resources and resourcefulness. Second, patients with low resourcefulness have difficulty learning stoma care skills, anticipating and managing problems such as leakage, and actively obtaining information and emotional support. Therefore, they cannot alleviate the distress caused by fecal incontinence and social embarrassment (44). This lack of coping ability is associated with greater psychological burden and social withdrawal, which are in turn related to a multidimensional decline in quality of life (45). In contrast, patients with higher resourcefulness can more actively manage stoma-related problems and mobilize social resources, thereby partially offsetting the negative impact of stress on quality of life (46).

In summary, perceived stress is heterogeneous among colorectal cancer patients with intestinal ostomies. Resourcefulness is consistent with a mediating role in the relationship between perceived stress and quality of life. Long−term high tension or loss of control weakens patients’ confidence in independent problem−solving and their willingness to seek help. This makes it difficult for them to effectively learn stoma care skills and obtain support (47), which in turn exacerbates defecation concerns, social avoidance, and psychological distress, ultimately impairing quality of life (16). Conversely, patients with high resourcefulness actively mobilize resources and buffer the impact of stress. According to the stress−coping theory, an individual’s cognitive appraisal of stressful events (perceived stress) dynamically shapes the accessibility and effectiveness of their coping resources (resourcefulness), which in turn determines quality of life through coping behaviors (9). Therefore, perceived stress may affect resourcefulness by altering how individuals appraise their own coping resources. This may, in turn, reduce patients’ ability to take proactive coping behaviours when facing stoma-related difficulties, which is further associated with diminished quality of life. This could be a potential mechanism underlying the mediating role of resourcefulness.

### Clinical implications

Our findings generate several preliminary hypotheses for future research. Notably, the Control-Loss Dominant Profile was the largest group (52.59%). This suggests that loss of control may be more central to ostomy-related distress than tension alone. Patients in this group may benefit from interventions that restore a sense of mastery—for example, structured self-care training and problem-solving skills. In contrast, patients in the Tension-Activated Profile may respond better to arousal-reduction strategies, such as relaxation techniques or mindfulness. For the Chronic High-Stress Profile, routine screening for psychological distress is recommended. Clear referral pathways to mental health services should be established. Psychosocial rehabilitation programs focusing on daily functioning and social participation may be considered. Family caregivers should be involved in the rehabilitation process. Psychoeducational and problem-solving interventions designed to enhance resourcefulness are also promising. However, these implications are preliminary and derived from cross-sectional data; they require testing in future interventional studies.

## Limitations

The present study has several limitations. First, the cross-sectional design precludes causal inference; therefore, the temporal sequence postulated by the Transactional Model of Stress and Coping cannot be empirically verified. Second, participants were recruited from only two tertiary hospitals in the Sichuan-Chongqing region via convenience sampling, which limits generalizability to some extent. Third, all data were self-reported, raising the potential for recall and social desirability bias; additionally, Harman’s single-factor test is limited for detecting common-method bias. Fourth, although LPA demonstrated good classification accuracy (entropy = 0.941), classification uncertainty was not fully incorporated into downstream analyses. Future studies may consider employing three-step approaches to account for classification error. Fifth, the three-profile solution requires replication in independent samples. Sixth, we did not collect data on disease stage, pain intensity, history of psychological disorders, treatment status, time since surgery, or ostomy duration, which may confound the observed associations. Given the early post-surgery time point (within one month), factors such as adjuvant chemotherapy or recurrence status were not applicable. Future studies with extended follow-up should address these factors. Finally, cultural factors may influence stress perception and resource utilization, highlighting the need for cross-cultural validation.

## Conclusions

This study identified three distinct perceived stress profiles among colorectal cancer patients with intestinal ostomy: Tension-Activated, Control-Loss Dominant, and Chronic High-Stress. Among these, the Chronic High-Stress Profile exhibited the lowest levels of resourcefulness and quality of life, whereas the Tension-Activated Profile had the highest. Resourcefulness is consistent with a partial mediating role in the relationship between perceived stress profiles and quality of life. These findings suggest that tailored interventions targeting specific stress profiles and enhancing resourcefulness may be promising and should be tested in longitudinal or interventional studies.

## Data Availability

Restrictions apply to the availability of these data, which were used under a data-sharing agreement in the present study and are therefore not publicly available. The data are, however, available upon reasonable request from the corresponding author. Requests to access the datasets should be directed to Xiaocui Zou, Email: zou_xiaocui@qq.com.

## References

[1] Sung H Ferlay J Siegel RL Laversanne M Soerjomataram I Jemal A . Global cancer statistics 2020: GLOBOCAN estimates of incidence and mortality worldwide for 36 cancers in 185 countries. CA Cancer J Clin. (2021) 71:209–49. doi: 10.3322/caac.21660 33538338

[2] Xia C Dong X Li H Cao M Sun D He S . Cancer statistics in China and United States, 2022: profiles, trends, and determinants. Chin Med J (Engl). (2022) 135:584–90. doi: 10.1097/CM9.0000000000002108 35143424 PMC8920425

[3] Zheng RS Chen R Han BF Wang SM Li L Sun KX . Cancer incidence and mortality in China, 2022. Chin J Oncol. (2024) 46:221–31. doi: 10.3760/cma.j.cn112152-20240119-00035 38468501

[4] Krouse RS Grant M McCorkle R Wendel CS Cobb MD Tallman NJ . A chronic care ostomy self-management program for cancer survivors. Psychooncology. (2016) 25:574–81. doi: 10.1002/pon.4078 26804708 PMC4833624

[5] Zhang HL Sun L Yuan L Shen MJ Chu AQ . Enterostomy nursing from the perspectives of bibliometrics and social network analysis. Mod Clin Nurs. (2019) 18:51–7. doi: 10.3969/j.issn.1671-8283.2019.08.010

[6] Jin DA Gu FP Meng TL Zhang XX . Effect of low anterior resection syndrome on quality of life in colorectal cancer patients: a retrospective observational study. World J Gastrointest Surg. (2023) 15:2123–32. doi: 10.4240/wjgs.v15.i10.2123 37969698 PMC10642465

[7] Chen YW He HJ Zhang Y Song JL . Chain mediating effect of stress perception and positive coping on nursing students' sense of meaning in life and professional identity. J Nurs Sci. (2023) 38:85–9. doi: 10.3870/j.issn.1001-4152.2023.17.085

[8] Jin YA Li CY Lin ZM Jin HH Cui YH Li XX . Living experience of patients with permanent enterostomy after surgery: a qualitative study. Chin J Gerontol. (2019) 39:3816–21. doi: 10.3969/j.issn.1005-9202.2019.15.067

[9] Li Y Liu CE Song QF . Study on perceived stress and its influencing factors of patients with enterostomy. J Nurs Train. (2018) 33:593–7. doi: 10.16821/j.cnki.hsjx.2018.07.005

[10] Kikuchi N Nishiyama T Sawada T Wang C Lin Y Watanabe Y . Perceived stress and colorectal cancer incidence: the Japan Collaborative Cohort Study. Sci Rep. (2017) 7:40363. doi: 10.1038/srep40363 28091607 PMC5238416

[11] Li L Shen YW Li DL Cheng FF Yu XF Pan LL . Analysis of influencing factors and pathways of postoperative self-efficacy in patients with fragility fracture. Chin J Nurs. (2024) 59:3003–8. doi: 10.3761/j.issn.0254-1769.2024.24.009

[12] Hu X Zhang Q Wang H Tan J Geng Q Ma H . Psychological distress among colorectal cancer patient-caregiver dyads: influencing factors and dyadic intervention preferences-a mixed methods study. Psychooncology. (2025) 34:e70287. doi: 10.1002/pon.70287 40973983

[13] Brivio E Guiddi P Scotto L Giudice AV Pettini G Busacchio D . Patients living with breast cancer during the coronavirus pandemic: the role of family resilience, coping flexibility, and locus of control on affective responses. Front Psychol. (2021) 11:567230. doi: 10.3389/fpsyg.2020.567230 33519580 PMC7840521

[14] Liu YY Wang YC Yu Y Zhang XP . Effects of structural and psychological empowerment interventions on disease-perceived stress, family support and self-efficacy in elderly patients with secondary stroke. China J Health Psychol. (2025) 33:1559–63. doi: 10.13342/j.cnki.cjhp.2025.10.022

[15] Duan BX . Status quo and influencing factors of resilience in patients with lung cancer. Chin Evid Based Nurs. (2023) 9:2452–5. doi: 10.12102/j.issn.2095-8668.2023.13.034

[16] Costa ALS Heitkemper MM Alencar GP Damiani LP Silva RMD Jarrett ME . Social support is a predictor of lower stress and higher quality of life and resilience in Brazilian patients with colorectal cancer. Cancer Nurs. (2017) 40:352–60. doi: 10.1097/NCC.0000000000000388 27171810

[17] Wang M Hanges PJ . Latent class procedures: applications to organizational research. Organ Res Methods. (2011) 14:24–31. doi: 10.1177/1094428110383988

[18] Bekhet AK . Self-assessed health in caregivers of persons with autism spectrum disorder: associations with depressive symptoms, positive cognitions, resourcefulness, and well-being. Perspect Psychiatr Care. (2014) 50:210–7. doi: 10.1111/ppc.12046 24206628

[19] Yu CX Li XL . Effect of mindfulness cancer rehabilitation training combined with wisdom theory intervention on stress perception and mood state in patients with advanced lung cancer undergoing chemotherapy. Chin Mod Med. (2025) 32:179–83. doi: 10.3969/j.issn.1674-4721.2025.16.038

[20] Guo YR Liu YJ Guo LN Wang J Yu SY Zhu YR . The mediating effect of resourcefulness between perceived pressure and depression in newly diagnosed breast cancer patients. Chin J Nurs. (2019) 54:1197–201. doi: 10.3761/j.issn.0254-1769.2019.08.016

[21] Kobiske KR Bekhet AK Garnier-Villarreal M Frenn M . Predeath grief, resourcefulness, and perceived stress among caregivers of partners with young-onset dementia. West J Nurs Res. (2019) 41:973–89. doi: 10.1177/0193945918806689 30343648

[22] Folkman S Lazarus RS . Stress, Appraisal, and Coping. New York: Springer Pub. Co (1984). p. 445.

[23] Folkman S Lazarus RS Dunkel-Schetter C DeLongis A Gruen RJ . Dynamics of a stressful encounter: cognitive appraisal, coping, and encounter outcomes. J Pers Soc Psychol. (1986) 50:992–1003. doi: 10.1037//0022-3514.50.5.992 3712234

[24] Li JW Wang SJ Li CZ Hu BS Jiao GM Zhang WH . Effects of nursing intervention based on stress and coping theory on psychological stress, negative emotions and self-management in patients with permanent enterostomy after surgery for colorectal cancer. J Nurs. (2025) 32:64–9. doi: 10.16460/j.issn1008-9969.2025.04.064

[25] Li J Yao H Lu Y Zhang S Zhang Z . Chinese national clinical practice guidelines on prevention, diagnosis and treatment of early colorectal cancer. Chin Med J (Engl). (2024) 137:2017–39. doi: 10.1097/CM9.0000000000003253 39104005 PMC11374253

[26] Cohen S Kamarck T Mermelstein R . A global measure of perceived stress. J Health Soc Behav. (1983) 24:385–96. doi: 10.2307/2136404 6668417

[27] Yang TZ Huang HT . An epidemiological study on stress among urban residents in social transition period. Chin J Epidemiol. (2003) 24:11–5. 14521764

[28] Zauszniewski JA Lai CY Tithiphontumrong S . Development and testing of the resourcefulness scale for older adults. J Nurs Meas. (2006) 14:57–68. doi: 10.1891/jnum.14.1.57 16764178

[29] Wang SM Lai CY Chang YY Huang CY Zauszniewski JA Yu CY . The relationships among work stress, resourcefulness, and depression level in psychiatric nurses. Arch Psychiatr Nurs. (2015) 29:64–70. doi: 10.1016/j.apnu.2014.10.002 25634877

[30] Prieto L Thorsen H Juul K . Development and validation of a quality of life questionnaire for patients with colostomy or ileostomy. Health Qual Life Outcomes. (2005) 3:62. doi: 10.1186/1477-7525-3-62 16219109 PMC1274339

[31] Shao L Lv L Zheng MC Huang MR Zhang JE . Adaptation and psychometric evaluation of the Stoma-QOL questionnaire among Chinese rectal cancer patients with colostomy. Int J Nurs Pract. (2022) 28:e13045. doi: 10.1111/ijn.13045 35274411

[32] Adhikari M Devkota HR Cesuroglu T . Barriers to and facilitators of diabetes self-management practices in Rupandehi, Nepal- multiple stakeholders' perspective. BMC Public Health. (2021) 21:1269. doi: 10.1186/s12889-021-11308-4 34187461 PMC8243465

[33] Kim SY . Determining the number of latent classes in single- and multi-phase growth mixture models. Struct Equ Modeling. (2014) 21:263–79. doi: 10.1080/10705511.2014.882690 24729675 PMC3979564

[34] Kang SJ Cho YR Park GM Ahn JM Han SB Lee JY . Predictors for functionally significant in-stent restenosis: an integrated analysis using coronary angiography, IVUS, and myocardial perfusion imaging. JACC Cardiovasc Imaging. (2013) 6:1183–90. doi: 10.1016/j.jcmg.2013.09.006 24229771

[35] Liang Q Wang BJ Liu Y Zhang TY Feng ZH Zhang CM . Latent profile analysis and influencing factors of resourcefulness in colostomy patients with colorectal cancer. Nurs Res. (2024) 38:3621–7. doi: 10.12102/j.issn.1009-6493.2024.20.009

[36] Hu H Dou W Qian M Wang Y . Latent profile analysis and influence factors of self-compassion among colorectal cancer patients with enterostomy from China: a cross-sectional study. BMJ Open. (2025) 15:e097955. doi: 10.1136/bmjopen-2024-097955 40983585 PMC12458851

[37] Lin H Lin R Yan M Lin L Sun X Wu M . Associations between preparedness, perceived stress, depression, and quality of life in family caregivers of patients with a temporary enterostomy. Eur J Oncol Nurs. (2024) 70:102557. doi: 10.1016/j.ejon.2024.102557 38581900

[38] Wang F Huang L Zhang H Jiang H Chang X Chu Y . The mediating role of perceived stress on the relationship between perceived social support and self-care ability among Chinese enterostomy patients. Support Care Cancer. (2021) 29:3155–62. doi: 10.1007/s00520-020-05829-8 33074359

[39] Li QE Zhao L Zeng HJ Luo M Fan YX Xie Y . Potential post-traumatic growth profile and its relationship with quality of life in patients with enterostomy. Mod Clin Nurs. (2023) 22:17–22. doi: 10.3969/j.issn.1671-8283.2023.05.003

[40] Hadrévi J Myte R Olsson T Palmqvist R Slunga Järvholm L Van Guelpen B . Work-related stress was not associated with increased cancer risk in a population-based cohort setting. Cancer Epidemiol Biomarkers Prev. (2022) 31:51–7. doi: 10.1158/1055-9965.EPI-21-0182 34697056 PMC9398123

[41] Xu CJ Xu PL Pan AH Wang Y Wu M Liu R . Latent profile analysis and nursing implications of emotional inhibition in stoma patients with colorectal cancer. Chin J Nurs. (2025) 60:1295–301. doi: 10.3761/j.issn.0254-1769.2025.11.003

[42] Jin Y Li X Ma H Xiong L Zhao M Wang H . Dyadic effects of perceived stress, relationship satisfaction and distress disclosure on emotional distress in colorectal cancer patients and their family caregivers: an actor-partner interdependence mediation model. Asia Pac J Oncol Nurs. (2024) 11:100580. doi: 10.1016/j.apjon.2024.100580 39351017 PMC11440261

[43] Soelling SJ Rubio-Chavez A Baird L Brindle ME Cooper Z Vranceanu AM . Adaptation and recovery challenges after ostomy surgery: qualitative study of clinician perspectives. J Surg Res. (2026) 318:154–61. doi: 10.1016/j.jss.2025.12.009 41529388

[44] Wen Y Cai SJ Chen XL . Application value of psychological intervention based on resourcefulness theory in patients with permanent enterostomy. China J Health Psychol. (2022) 30:1169–75. doi: 10.13342/j.cnki.cjhp.2022.08.011

[45] Galicia Pacheco SI Petrova D Garrido D Catena A Madrid Pérez-Esparza B Torralba C . The relationship between psychological stress and cancer incidence: a systematic review and meta-analysis. Health Psychol Rev. (2025) 20:1–27. doi: 10.1080/17437199.2025.2590491 41332309

[46] Liu J Xu Y Song M Li X . The role of family care on the quality of life of patients with permanent enterostomies: a chain-mediated effect of inner strength and patient activation. Scand J Caring Sci. (2026) 40:e70226. doi: 10.1111/scs.70226 41872709 PMC13009318

[47] Ayaz-Alkaya S . Overview of psychosocial problems in individuals with stoma: a review of literature. Int Wound J. (2019) 16:243–9. doi: 10.1111/iwj.13018 30392194 PMC7948730

